# Neurocognitive outcomes of individuals with a sex chromosome trisomy: XXX, XYY, or XXY: a systematic review*

**DOI:** 10.1111/j.1469-8749.2009.03545.x

**Published:** 2010-01-05

**Authors:** VICTORIA LEGGETT, PATRICIA JACOBS, KATE NATION, GAIA SCERIF, DOROTHY V M BISHOP

**Affiliations:** 1 Department of Experimental Psychology, University of OxfordUK; 2 Wessex Regional Genetics Laboratory, Salisbury District HospitalSalisbury, Wiltshire, UK; 3 Division of Human Genetics, Southampton General HospitalSouthampton, Hampshire, UK

## Abstract

**Aim:**

To review systematically the neurodevelopmental characteristics of individuals with sex chromosome trisomies (SCTs).

**Method:**

A bibliographic search identified English-language articles on SCTs. The focus was on studies unbiased by clinical referral, with power of at least 0.69 to detect an effect size of 1.0.

**Results:**

We identified 35 articles on five neonatally identified samples that had adequate power for our review. An additional 11 studies were included where cases had been identified for reasons other than neurodevelopmental concerns. Individuals with an additional X chromosome had mean IQs that were within broadly normal limits but lower than the respective comparison groups, with verbal IQ most affected. Cognitive outcomes were poorest for females with XXX. Males with XYY had normal-range IQs, but all three SCT groups (XXX, XXY, and XYY) had marked difficulties in speech and language, motor skills, and educational achievement. Nevertheless, most adults with SCTs lived independently. Less evidence was available for brain structure and for attention, social, and psychiatric outcomes. Within each group there was much variation.

**Interpretation:**

Individuals with SCTs are at risk of cognitive and behavioural difficulties. However, the evidence base is slender, and further research is needed to ascertain the nature, severity, and causes of these difficulties in unselected samples.

Errors in the process of maternal or paternal meiosis can lead to chromosome trisomies. Among the most common trisomies compatible with live birth are those involving the X and Y chromosomes. Early studies established that the frequency of each of the sex chromosome trisomies (SCTs), XXY (Klinefelter syndrome), XXX, and XYY, was about one in 1000 same-sex individuals.^1^–^3^ In a more recent analysis of data from newborn surveys, the prevalence of XXY rose from 1.09 to 1.72 per 1000 male births, whereas the frequency of the other two SCTs remained stable.^4^

Although SCTs are not rare, there is a scarcity of rigorous evidence on prognosis and cognitive outcomes. One reason is that SCTs are not associated with gross physical or cognitive impairments and so may go unidentified. A corollary of this is that children who are identified may be atypical, with the SCT discovered only after clinical referral for cognitive or behavioural difficulties.^5^ This introduces potential distortion into the research literature if samples include children ascertained only after clinical referral.

There are three reasons why it is important to obtain an unbiased picture of the cognitive and behavioural outcomes of children with SCTs. First, SCTs can be identified on prenatal screening for other conditions, and parents might be offered termination of pregnancy on this basis. Those counselling them need accurate information about the range of outcomes that might be expected, in order to offer objective advice to parents.^6^ Second, if a child with a SCT is found to have developmental difficulties, clinicians need information to advise on prognosis and to offer early intervention, where appropriate. Third, children with SCTs can inform our understanding of how sex chromosomes affect neurodevelopment.^7^

Our aim in this review is to synthesize what is known about neurocognitive and behavioural outcomes in individuals with the XXY, XXX, or XYY karyotype, and to highlight areas where there are gaps in existing knowledge. For each karyotype, the research literature will be evaluated for information in five domains of development: general intelligence; scholastic strengths and weaknesses; attention and executive control; motor skills, speech and language; and social communication, interaction, and adaptation. We also review what is known about the risk of psychopathology and neurological correlates associated with each karyotype.

## Method

We conducted a systematic review of the past five decades of published data on cognitive and behavioural outcomes of individuals with an extra sex chromosome. The data were obtained primarily through published articles, although authors were contacted for clarification where appropriate. The online Web of Knowledge database was used to identify English-language articles, using the search terms (XXX OR XXY OR XYY OR sex chromosome (trisomy OR anomaly OR abnormality OR aneuploidy) OR Klinefelter) AND (cognit* OR language OR behav* OR brain OR neuro*). The search was confined to articles in databases for genetics, neuroscience, behavioural science, and psychiatry. Further articles were traced through PubMed and from reference lists in identified papers.

**Figure 1:**
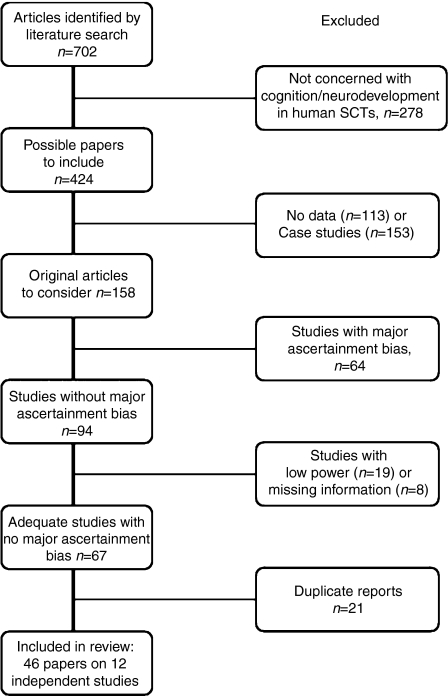
Procedure for selection of articles on sex chromosome trisomies (SCTs) for inclusion in the review.

Because letter combinations such as XXX are used with various meanings in other disciplines, the search criteria generated many irrelevant papers, but these were readily identified, leaving 702 papers with a focus on chromosome abnormalities. As shown in Fig. 1, we excluded articles describing animal studies, those dealing with conditions other than SCTs, and those with no cognitive or neurodevelopmental content, leaving a pool of 424 publications to evaluate. Of these, 113 did not include new empirical data, and 153 were case reports.

As we aimed to characterize the phenotype in as unbiased a way as possible, we next excluded articles with major ascertainment bias (*n*=64), that is, studies of individuals recruited through institutions for cognitive or psychiatric disorders, as well as those who were identified via patient support groups or through postnatal chromosome testing. Our rationale was that it was not usually possible to tell why a chromosome test had been conducted, or why an individual had joined a support group. Thus, we erred on the side of caution and excluded those who may have come to attention because of cognitive or behavioural difficulties. However, we did not exclude males with XXY who were identified as adults because of infertility or endocrinological concerns, because there is no reason to suppose that they were cognitively atypical. Studies of a sample of adults identified via population chromosome screening of tall males were also included.

Having identified 94 studies without major ascertainment bias, we next excluded studies that did not meet the following methodological inclusion criteria: (1) power of at least 0.69 to detect an effect size (Cohen’s d) of at least 1.0 on a one-tailed test, computed using G*Power 3;^8^ for categorical data, this effect size corresponded to an odds ratio of 6.11;^9^ and (2) sufficient information provided to evaluate power and other key methodological points. To avoid excluding most studies, the power criterion had to be set lower than is typically used; in effect it meant that studies were excluded unless the total sample (one SCT group plus any comparison groups) was at least 20. Where no comparison group of typically developing individuals had been assessed, the study was included if the sample size for a given karyotype was >10 and if well standardized measures had been used, allowing an estimation of performance relative to a comparable normative sample. In addition, to boost information on XYY cases from an unbiased source, two studies including more than 12 prenatally ascertained cases of at least one karyotype were included,^5^,^10^ despite lack of normal comparison groups or standardized measures.

Overall, 46 articles met our criteria (see Tables SI and SII,^11^,^54^ published online), but these were reports of data from only 12 independent samples. Most samples came from state- or countrywide screens of newborn infants initiated in the 1950s to 1960s, of which five – based in Boston and Denver in the USA, Edinburgh in the UK (two surveys), and Toronto in Canada – had sample sizes for at least one SCT that met our power criterion. Studies are grouped by cohort in Table SI. Of the 46 articles, 35 are descriptions of data from cases identified neonatally. The remaining articles comprise (1) four reports on a group of males with XXY and XYY identified in Copenhagen, Denmark, by screening of adult males from the general population who were 184cm tall or more; (2) five studies from four cohorts of males with XXY that met our power criterion where, although the diagnosis was made postnatally, this was prompted by concerns about physical development or fertility in adulthood, rather than cognitive or behavioural difficulties; and (3) the two aforementioned uncontrolled studies of children identified on prenatal screening. Where it appeared that there was duplication or overlap in data reported in different papers, only the publication with the largest sample is considered.

When reviewing results from this literature, a *p* value of <0.05 was regarded as statistically significant. Where relevant, we report effect sizes, to provide a standardized measure of difference between two groups. This can be more informative than significance levels, especially when considering the clinical importance of deficits.

## Results

### XXY karyotype (Klinefelter syndrome)

#### General intelligence

**Figure 2:**
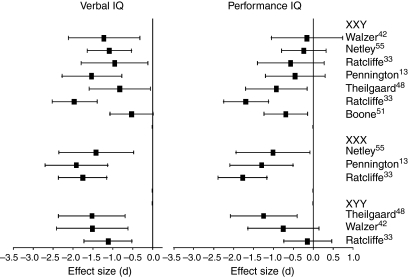
Effect sizes for comparisons of Wechsler IQ in individuals with or without sex chromosome trisomies (SCTs).

In Fig. 2 the effect sizes on verbal and performance IQ are summarized for studies using Wechsler IQ measures. Where IQ was reported for the same cohort in different articles, we selected the one with the largest sample size. Theilgaard^48^ did not report SDs, and a value of 15 was assumed so that we could compute effect size. On average, verbal IQ of males with XXY was 18 points lower than that of comparison groups, and performance IQ was 11 points lower. However, a notable feature of most of these studies is the high ability of individuals in the comparison groups, who were usually well matched on socio-economic status: for the studies shown in Fig. 2 the weighted mean values for the comparison groups was 112 for verbal IQ and 111 for performance IQ. Although depressed relative to the comparison groups, the IQs of the XXY males were generally within normal limits: weighted mean values are 95 and 100 for verbal and performance IQ respectively, for those included in Fig. 2.

Netley^55^ collated Wechsler IQ data from several studies of newborn infants, including some with too few cases for independent analysis, and found that at an average age of 10 years, mean verbal IQ was 90.11 (SD 17.71) in 73 males with XXY and 102.4 (SD 14.4) in 60 unaffected individuals, which is a significant difference, with effect size of 0.76. For performance IQ the means were 102.6 (SD 13.11) for the XXY group and 104.44 (SD 14.44) for the comparison group, which is a non-significant difference. The Edinburgh cohort was excluded from this composite analysis because of the high IQ of the comparison group.

The overall picture from these analyses is that verbal IQ is depressed in relation to the level expected from social background, but it is nevertheless usually within low normal limits, whereas performance IQ is relatively unimpaired.

#### Scholastic strengths and weaknesses

In all surveys of unbiased samples, the authors have reported increased educational difficulties in males with an extra X chromosome, regardless of whether this is defined in terms of a specific learning disability (11 of 13 cases in the Boston sample^42^) or provision of special help at school (11 of 14 cases in the Denver neonatal sample,^15^ eight of 14 cases in the Denver prenatal sample,^10^ 77% in the Edinburgh sample,^33^ 4/10 in the Toronto sample^41^). Consistent with this, males with XXY tended to leave school earlier and achieved a lower educational level than unaffected males.^23^,^46^ There is, however, evidence of variability within the group. In a survey of parents of prenatally diagnosed children aged 7 to 18 years, Linden and Bender^10^ found that, although reading was reported to be a difficulty for eight of 14 males with an extra X chromosome, for five it was a relative strength. However, these data must be considered with caution because of the possibility of biased reporting by parents and the lack of an appropriate comparison group; this was a self-selected sample of parents who had sought advice from the Denver team after obtaining a prenatal diagnosis, and most came from upper socio-economic backgrounds. Information was obtained from an unstandardized questionnaire with no normative data. Nevertheless, the authors noted that their paper provided the only published information about outcomes for a prenatally diagnosed group.

The same group reported relatively good arithmetic skills in males with XXY, but once again they relied on parental report, rather than formal testing.

Direct studies of educational skills confirmed an average deficit in reading accuracy and reading comprehension for males with XXY relative to unaffected males, with a large effect size (*d*>1).^13^,^24^,^41^,^44^ Fewer data are available on mathematics, but in general results suggest that this was less of a difficulty than reading, although mathematical ability tended to be lower than in comparison groups.^24^,^41^,^51^^.^

Despite a high rate of specific learning disability, in samples followed to adulthood, some individuals did well at school and achieved university degrees.^24^,^33^

#### Attention and executive control

Pennington et al.^13^ categorized school-aged children from the Denver neonatal sample according to clinical judgement of specific learning difficulties. In contrast to other types of learning difficulty, the rate of attention deficit disorder (four of 15 cases) was no higher than in unaffected siblings (four of 27 cases). In their uncontrolled study of prenatally diagnosed cases, Linden and Bender^10^ reported that three of 14 males had a diagnosis of attention deficit disorder, and a further four were noted by parents to be distractible.

Only a handful of research groups used neuropsychological tests to assess attention and executive function directly. Theilgaard^48^ found that males with XXY were significantly slower than an age-matched comparison group on the Stroop Colour-Naming test, but they were unimpaired compared with an unaffected group matched for IQ as well as age. They did not differ significantly from either comparison group on the Matching Familiar Figures test. These two tests have been used to index problems with inhibition and impulsivity respectively, although their validity and relevance for assessing attention deficit has been queried.^56^,^57^ In adulthood, males with XXY from the Denver neonatal cohort did not differ from comparison groups on the Wisconsin Card Sorting Task, an executive task to assess cognitive flexibility and planning.^24^ Boone et al.^51^ administered a neuropsychological battery that included verbal and nonverbal executive tasks.

Overall, the limited data available suggest that any attentional or executive deficits in XXY males are consistent with general ability, rather than indicative of disproportionate difficulties in these areas.

#### Motor skills

A study of the Toronto newborn sample used population norms to identify impairment on the revised Yale Developmental Inventory in toddlers. A deficit in gross motor skills was found in 11 of 25 males with XXY, and a deficit in fine motor skills was found in seven of the 25 males.^35^ In a detailed study of the Denver cohort focusing on motor development, Salbenblatt et al.^17^ used a range of standardized tests together with clinical examination and rated 11 of 14 males as dysfunctional. Symptoms included hypotonia, primitive tonic neck reflex, hand tremor, poor strength, and hypermobility of fingers and elbows. Eight of 14 males with XXY had started walking at a late age and, on a quantitative measure of sensorimotor integration administered at 6 to 15 years of age they performed significantly worse than comparison groups, with an effect size of 1.1SD. The Bruininks–Oseretsky Test of Motor Proficiency revealed significant deficits in gross motor skills (*d*=0.7), but only a trend for impairment in fine motor skills (*d*=0.3). Motor deficits were also seen in adults, with Boone et al.^51^ finding a mean difference of 1.05SD on speeded peg moving between males with XXY and comparison groups.

Although it has been claimed that there is a high rate of non-right-handedness in males with XXY karyotype,^38^ this was not seen in the Edinburgh or Copenhagen samples.^29^,^48^^.^

#### Speech and language

Significant delays in the earliest stages of language development, assessed by developmental schedules, were reported in the Edinburgh,^28^ Boston,^42^ Denver,^11^ and Toronto^35^ neonatal cohorts. Subsequently, 53% of the Denver sample received speech and language therapy,^13^ and 68% were judged by a speech and language therapist to have a language impairment at around 13 years of age.^16^ A rather lower proportion, four of 14 males, were reported to have had intervention for speech and language difficulties in the Denver prenatal sample.^10^ A range of language tests has been used for direct assessment of males with XXY, and in general these have confirmed the presence of impairments identified by parental reports, especially on tests that tax expressive language.^14^,^28^,^37^,^44^,^51^ In the most comprehensive study of language in males with XXY,^44^ Graham et al. found significant differences between 14 males with XXY and 15 unaffected males aged 5 to 12 years on tests of verbal expression (*d*=0.7–0.8) and verbal memory (*d*=1.1–1.2). Differences in receptive language were mostly non-significant, but effect sizes were around 0.6, suggesting that they might be reliable in a larger sample.

#### Social communication, interaction, and adaptation

Children from the Edinburgh cohort did not differ from comparison groups on the Behaviour Screening Questionnaire at age 3 years,^30^ and the Toronto neonatal cohort (*n=*24) did not differ at age 6 years on the Vineland Social Maturity Scale.^36^ In the Boston neonatal sample,^43^ males with XXY were directly observed at home in a longitudinal study and were found to score lower than comparison groups on measures of activity level, intensity of response, and approach to new events, as well as showing higher pliancy. No difference was observed in social relationships. The authors commented that the passive, withdrawn behavioural style of these males led to their being offered less educational support than other children who were more disruptive. This fits with a characterization on a teacher report (Bristol Social Adjustment Guide) of children from the Edinburgh cohort as significantly ‘under-reactive’ at 7 to 10 years of age compared with unaffected children.^30^ Their mean score was around the 30th centile. In adolescence, males in the Denver neonatal cohort were described as ‘characteristically reticent and lacking in confidence’, but most had friends and dated females.^20^ By their mid-30s many were in stable relationships, and all but one of the 11 males in the Denver sample was employed.^23^ Schiavi et al.^49^ found that males with XXY were later than comparison groups to gain their first sexual experiences.

Taken together, these studies suggest that males with XXY do not usually have major problems with social interaction and adaptation, although they may be timid and unassertive.

#### Risk of psychopathology

Götz et al.^32^ interviewed 13 males from the Edinburgh neonatal survey and 45 unaffected males of comparable socio-economic status when they were around 20 years of age. None met diagnostic criteria for a major psychiatric disorder. The males with XXY showed increased antisocial behaviour in adolescence (odds ratio 6.2) and a more unstable occupational history (odds ratio 4.5), but a minority met criteria for antisocial behaviour disorder in adulthood (three of 13 vs six of the 45 males in the comparison group), and the number with criminal convictions was similar to that in the comparison group (12/100 in both groups). Ten males with XXY from this cohort who participated in a brain-imaging study did not differ from the comparison group on a structured interview for schizotypy.^34^ Males from the Denver cohort did not differ from their siblings in global measures of mental health in adolescence,^20^ in young adulthood,^21^ and by their mid-30s.^23^

#### Neural correlates

*Overall brain volumetry.* It has been suggested that the lower IQ in males with XXY may be related to reduced brain growth, given that head circumference during childhood is smaller than that of unaffected individuals, despite greater height (*d*≈1).^31^ Nevertheless, those investigators did not find that head size predicted IQ in males with XXY. Subsequent studies using brain imaging have shown reduced brain volume in adults with XXY relative to XY males,^25^,^34^ although these studies, each of a neonatally identified sample, were based on only 10 XXY cases each, and the difference reached significance in only one (*d*=1.6). In a further study of 18 adults being treated for hypogonadism, the investigators did not find any difference in brain volume compared with unaffected males who were closely similar in IQ.^53^

It has been suggested that increases in ventricle volume are associated with the XXY karyotype,^34^,^53^ and that ventricle volume is inversely correlated with verbal executive functions (*r*=−0.51, *p*=0.003), as well as with overall verbal processing speed.^53^ The increased lateral ventricle volume, in conjunction with a reduction in total cerebral volume, suggests reduced volume of both white and grey matter in males with XXY.

*Left-hemisphere structure and function.* There has been a long-standing interest in the notion that language deficits in individuals with SCTs may be linked to atypical cerebral lateralization, starting with a study by Netley and Rovet^39^ who found smaller left-hemisphere asymmetries and larger right-hemisphere asymmetries on behavioural tasks in males with XXY than in unaffected males. Potentially, a reduction in left-hemisphere specialization may have an effect on, or be a consequence of, an individual’s ability to manipulate and understand language. One structural study revealed significant decreases in left, but not right, temporal grey matter, with an effect size 1.29.^22^ This structural difference may also be related to reported differences in brain perfusion (and therefore function) of temporal regions in 27 patients with Klinefelter syndrome, for whom hypoperfusion was more frequent in the left than in the right hemisphere, and in temporal and temporoparietal areas.^52^ However, in the study by Warwick et al.^34^ the trend was for smaller volume in the right temporal lobe for those with XXY.

*Amygdala.* A brain basis for problems with socio-emotional development in XXY has been suggested in terms of a significant reduction in amygdala volume compared with comparison groups.^25^ The large effect size of this result (*d*=1.7) is all the more striking when compared with the results from hippocampal volume, which gave identical mean values in the XXY and comparison groups. However, the authors’ interpretation, linking this finding to risk of psychopathology, seems highly speculative, given that the participants came from the Denver cohort, who showed very little evidence of psychopathology in adulthood (see above).

*Cerebellum.*Little is known about the neurological origins of motor difficulties in Klinefelter syndrome. On magnetic resonance imaging, Itti et al.^53^ found significant bilateral reductions in cerebellar volume compared with the comparison group, but this was not correlated with the motor speed impairment that the males in their study experienced. Only non-significant trends were found for motor and brain correlates of testosterone treatment in this study.

*Brain function.* There is very little direct evidence relating to differences in brain function in individuals with SCTs. In the only electrophysiological study meeting our sampling criteria, the authors reported reduced amplitude of the P300 event-related potential component in a sample of 32 individuals with XXY compared with males with untreated idiopathic hypogonadotrophic hypogonadism and healthy males, and they construed this difference as a measure of overall central nervous system function.^54^

### XXX karyotype

#### General intelligence

Despite the small evidence base from only four cohorts of unselected females with an third X chromosome, data on IQ are consistent, indicating a significant reduction in IQ of approximately 20 points (see Fig. 2). Both verbal and performance IQ are affected, although, when data from several neonatal studies were combined to give 32 XXX and 25 unaffected females with a mean age of 10 years, Netley^55^ found that the deficit in verbal IQ (*d*=1.24) was significantly greater than that in performance IQ (*d*=1.05).

#### Scholastic strengths and weaknesses

All studies of literacy skills have shown significant reading impairments, with most females needing some extra assistance with reading (*d*>1).^10^,^13^,^16^,^24^,^33^ The finding that some females needed no specific assistance with reading^13^ does not necessarily mean that they had good reading skills; instead, this may reflect a generally depressed cognitive profile rather than specific reading disability. Studies concur in finding that females with XXX showed significant difficulties with arithmetic, with around 65% of females finding it difficult.^10^,^24^ Nevertheless, although most of the 11 XXX females from the Denver sample found school a struggle, two went on to obtain university degrees.^21^

#### Attention and executive control

In conjunction with depressed scholastic achievement, Pennington et al.^13^ and Linden and Bender^10^ reported higher levels of attention deficit in females with XXX than in siblings, along with increased levels of distractibility. These reports were based on either clinical judgements or parental report only; the attentional and executive profile of females with XXX was not investigated directly in any of the articles considered in this review.

#### Motor abilities

Like males with XXY, females with an extra X chromosome showed delayed motor development and late walking,^35^ with gross motor difficulties evident in females between the ages of 6 and 14 years.^12^,^19^ Salbenblatt et al.^19^ used quantitative standardized measures of sensorimotor integration and gross and fine motor skills, and they found significant deficits in all three areas, with effect sizes of *d*=3.2, 2.2, and 1.0 respectively. Some difficulties with motor planning were also observed.

Females with XXX have been reported as taking part in a variety of sports, including team sports, despite reports of reduced gross motor coordination.^10^,^19^

#### Speech and language

Difficulties with speech and language have been noted in females with an extra X chromosome, with 40 to 91% needing speech therapy at some point.^10^,^12^,^33^ In the Edinburgh, Denver, and Toronto cohorts, there was a clear language delay in the preschool years.^12^,^28^,^35^ Bender et al.^14^ found that speech and language therapists rated these females as having severe impairments in auditory processing (44/100), comprehension (56%), expression (78%), and speech (11/100). Pennington et al.^12^ administered the Illinois Test of Psycholinguistic Abilities to 11 4-year-old females from the Denver cohort and found that they scored below normal limits on auditory association (*d*=1.2), visual association (0.8), visual closure (0.7), and auditory closure (1.9). The presence of such pervasive difficulties with language skills suggests that the difficulties are not simply a secondary consequence of motor or articulatory limitations. However, this result must be interpreted with some caution because the comparison was with test norms rather than a matched comparison group.

#### Social communication, interaction, and adaptation

In middle childhood and adolescence, females with XXX appeared to develop good relationships, and were described as well liked by friends.^10^,^20^ They were, however, often rated as immature and as having poor social adjustment.^20^ This trend continued throughout adulthood where problems in social adjustment remained in some females. Sixteen females were followed into adulthood in the Edinburgh cohort and were described as gaining employment in non-academic occupations such as hairdressing or waitressing, with four being housewives. By their mid 30s, eight of 11 females in the Denver cohort had children of their own.^23^

#### Risk of psychopathology

Bender et al.^20^,^21^,^23^ looked at psychosocial outcomes in adolescence and adulthood for the Denver cohort. Females with XXX were found to have lower scores on a global assessment of functioning (*d*=2.1 in adolescence, *d*=1.6 in mid-20s) and eight of 11 adolescents had DSM-III-R^58^ diagnoses, with depression being the most common. By their mid-20s, these females continued to report relatively high rates of psychiatric symptoms compared with both their own comparison groups and other SCT groups, with phobic anxiety and interpersonal sensitivity being particular problems.^21^ Bender et al.^23^ reported, however, that by their mid-30s many females had overcome earlier problems, achieving greater independence and improved personal relationships.

One other study with some pertinent information is the brain-imaging study of the Edinburgh sample, where the Structured Interview for Schizotypy was administered to 11 females with the XXX karyotype and 13 unaffected females.^34^ Detailed data were not presented by the authors, but it was reported that the XXX females were over-represented among high scorers on introversion, magical thinking, and impulsivity. It is unclear, however, whether this was related to the lower IQs of the XXX females.

#### Neural correlates

Magnetic resonance imaging of brain volume has been conducted in 10 XXX females from the Denver cohort^25^ and 11 females from the Edinburgh neonatal cohort.^34^ In both studies whole-brain volumes were found to be significantly reduced in females with an extra X chromosome compared with comparison groups, with effect sizes of 1.48 and 1.36 respectively,^25^,^34^ but this was not significantly correlated with estimates of IQ from the National Adult Reading Test and Quick Test in the XXX group.^34^

The similarity between individuals with XXY and XXX has been suggested to indicate that ‘the presence of a supernumerary X chromosome has a demonstrable effect on brain development’^34^ and may lead to a reduction in total brain volume. However, unlike males with XXY, there was no significant difference in lateral ventricle volume relative to whole-brain volume between XXX females and unaffected females,^34^ suggesting that the presence of an extra X chromosome does not have the same impact in males and females.

Regional brain volumes and asymmetries did not differ significantly between females with the XXX karyotype and unaffected females after adjustment for whole-brain volume.^34^ In a study of the Denver sample focused specifically on the amygdala and related structures, the investigators found only a non-significant trend for smaller amygdala volume in females with XXX, and no difference in hippocampal volumes, once whole-brain volume had been corrected for.^25^

### XYY karyotype

#### General intelligence

As shown in Fig. 2, IQ was measured using Wechsler tests in three studies of males with XYY, all of whom had deficits in verbal IQ (effect size 1.0–1.5) relative to comparison groups. For the Theilgaard study,^48^ the SD had to be estimated as 15. Deficits were less marked in performance IQ and were non-significant in one study. Once again, it is important to note that these are relative deficits: in all studies, the comparison groups comprised males matched for socio-economic status, who achieved above-average levels of IQ. Thus, although males with XYY had lower IQ scores than expected for their social background, they were not impaired in relation to general population norms. Weighted means were 99.5 for XYY males and 114.9 for comparison groups on verbal IQ, and 106.4 and 114.8, respectively, on performance IQ.

Netley^55^ combined Wechsler IQ data for 28 XYY males from different neonatal surveys, excluding the Edinburgh cohort, and found no significant difference compared with eight unaffected males on either verbal IQ (XYY mean 100.7, SD 14.28, comparison group mean 104.5, SD 14.48) or performance IQ (XYY mean 108.79, SD 16.35, comparison group mean 104.6, SD 8.58).

#### Scholastic strengths and weaknesses

Witkin et al.^46^ created an educational index that reflected the number of national examinations passed and found that this was significantly lower in the 12 XYY males in their sample than in 4096 XY males who were screened because they were more than 184cm tall (*d*=0.9). The two groups did not differ in parental socio-economic status. This fits with the IQ data in suggesting that XYY males underachieve relative to their social background.

Ratcliffe^33^ summarized the progress of 19 males with XYY from the Edinburgh newborn sample and found that 54% were identified by teachers as having reading difficulties, compared with 18/100 of unaffected males in the comparison group. Difficulties with mathematics were not evident. She also noted that five males went on to university or technical college.

The only other information about educational progress in an unselected sample comes from Linden and Bender’s survey^10^ of parents of prenatally diagnosed males aged 8 to 16 years. This sample was skewed in favour of high socio-economic status, and there was no comparison group. In five of 14 cases, parents reported that their child had been given extra help for academic difficulties; in two cases the child was receiving special schooling. Nevertheless, nine males reported reading as a relative strength, and proficiency in science and mathematics was commented on.

#### Attention and executive control

Three of the 14 XYY males in Linden and Bender’s sample had a diagnosis of attention deficit disorder, and three more were described as distractible.^10^ Only one study described data from neuropsychological tests. Theilgaard^48^ compared 12 XYY males with 12 unaffected males matched on age and height and 12 unaffected males also matched on IQ.^48^ The XYY males did not differ from either comparison group on the Stroop test nor on the Matching Familiar Figures test but, as noted above, the validity of these tests for assessing executive functions has been questioned.

#### Motor abilities

Data on motor abilities were available for only four XYY males from the Denver newborn study; three of these males scored below the 10th centile on a composite score based on a battery of motor tests. In the Edinburgh newborn cohort, the XYY group scored 1.29 SD below the comparison group on a test of fine motor coordination, and 1.42 SD below the comparison group on balance.^33^

Delayed motor development or lack of motor coordination was also remarked on for five of 14 cases studied by Linden and Bender^10^ and three of the 12 XYY males in the prenatally diagnosed sample studied by Geerts et al.^5^ Both of these studies of prenatal samples are limited by lack of comparison groups and reliance on unstandardized parental reporting, but nevertheless they provide information about the frequency of parental concerns that may be of clinical use, given the general paucity of information on outcomes for this karyotype.

It is worth considering whether some of the cognitive and communicative difficulties affecting males with XYY may be related to motor impairments, but this has not been possible to establish, because of lack of data on different domains in the same individuals.

#### Speech and language

There is a dearth of information on speech and language development in males with an extra Y chromosome. In an uncontrolled study, Geerts et al.^5^ obtained information from a parental questionnaire about 12 prenatally diagnosed males with XYY at a mean age of 5 years. Half of the males had a marked delay in language development, and four of them had received speech and language therapy. This is consistent with findings from the Edinburgh newborn cohort, where 42% had delayed speech development, compared with 18/100 of unaffected males.^33^ However, in Linden and Bender’s prenatally diagnosed sample,^10^ only two of 14 XYY males had received speech and language therapy.

#### Social communication, interaction, and adaptation

Ratcliffe et al. found little evidence of behaviour difficulties in nine 3-year-olds with XYY, on the Behaviour Screening Questionnaire^59^ (same sample as Holsti^30^). Furthermore, friendships were not identified as particularly problematic: in Linden and Bender’s survey of prenatally diagnosed males,^10^ parents mostly described their children as well-liked and having friends. Nevertheless, problems with anger control were evident in childhood, with temper tantrums being identified as a feature in five of 14 XYY males.^10^ This is consistent with data from the Edinburgh cohort.^28^

Interview data from adults in the Copenhagen cohort^54^ provide some evidence that males with XYY were more aggressive than comparison groups towards their partners, although there was no support for the notion that XYY males were particularly violent or aggressive in general. In a separate study of sexuality in the same sample, males with XYY were less often married than comparison groups and expressed more sexual dissatisfaction.^49^

#### Risk of psychopathology

One of the first studies of XYY was conducted at a Scottish special hospital for individuals with mental health problems and behaviour dangerous to either themselves or to the public.^60^ The authors tested 197 of the hospital patients and found eight individuals with XYY; this was a more than 30-fold increase on the expected level. As noted by Bender and Berch,^61^ this led to an explosion in studies on institutionalized populations, with findings often incorrectly interpreted as indicating a higher prevalence of criminal behaviour in males with XYY, because people failed to take into account possible reasons why the males were in the hospital.

The first unbiased sample, recruited by Witkin et al.^46^ confirmed that rates of criminal conviction were indeed higher among 12 XYY males (41.7/100) than among 4098 XY males (9.3/100). However, as the authors noted, the crimes did not generally involve aggression, and part (although not all) of the difference in criminality between XYY and comparison groups could be accounted for by the lower intelligence of the XYY males: someone of lower IQ may be more easily led and may be more likely to be caught if he commits a crime.

This issue was further assessed by a detailed investigation of the Edinburgh newborn cohort in adulthood,^32^ in which a significantly higher proportion of males with XYY showed antisocial behaviour in adulthood than their peers (odds ratio 5.15). However, most cases fell short of clinical concern: in the XYY group, six of 16 males met research diagnostic criteria for antisocial behaviour disorder, compared with six of 45 unaffected males (a non-significant difference).

#### Neural correlates

A single study of 10 XYY males met inclusion criteria for this review.^34^ These males were found to have no structural differences from unaffected males, with preserved cerebral and ventricle volume.^34^

## Discussion

### Similarities and differences between the SCTs

Our review highlights that, although outcomes are variable, individuals with an extra sex chromosome are at risk of a range of neurodevelopmental difficulties. Deficits in IQ have been reported in those with an extra X chromosome, especially in females with XXX. Rates of speech and language impairments are elevated across all three groups, and difficulties with reading and educational achievement are often seen. Motor skills are impaired in all groups, and there is some suggestion of difficulties in attention and executive control, although this has barely been investigated. Although outcomes are variable, difficulties with social interaction, communication, and adaptation seem most apparent in females with XXX, who also showed an elevated risk of psychiatric disturbances such as anxiety and depression. Across all domains, most authors describe considerable variability within their samples, with some individuals performing in the normal or above-average range. The handful of studies on adult outcomes indicates that most individuals with a SCT are able to live independently and form normal adult relationships.

Although all three trisomies are characterized by an increased risk of educational problems, the specific profiles differ. Males with XYY have average IQs, consistent with the one study showing normal brain volume on magnetic resonance imaging. In contrast, both XXY and XXX groups tend to have below-average verbal IQ and small cerebral volume. However, whereas both verbal and performance IQ tend to be low in females with XXX, there is more selective impairment of verbal IQ in males with XXY. Neurological studies showed that ventricle volume was increased in males with XXY but not in females with XXX, whereas amygdala volume was significantly reduced only in males with XXY, once brain size had been taken into consideration.

Interpretation of such findings is complicated by uncertainty about robustness of results from small samples, and it is clear from our review that there are many contradictory findings in the literature, even in domains that have been assessed repeatedly, suggesting substantial heterogeneity within each of these groups.

### Methodological considerations

Our review has revealed a number of important limitations that characterize the existing literature. A serious issue is sample size, which constrains statistical power. To have sufficient studies to review, we needed to relax the power criterion for inclusion to 0.69 to detect an effect size of 1, whereas power is ordinarily set at a minimum of 0.8.^62^ Effect sizes smaller than 1 are likely to be of clinical significance but would have a low chance of being detected with these sample sizes. Small sample sizes also limit attempts to understand variability between individuals with SCTs and the risks and protective factors that may moderate outcomes (however, see the article by Bender et al.^16^).

Small study samples of children with SCTs are understandable when one considers that to obtain an unbiased sample one has to screen around 1000 children to identify a single case with XYY or XXX. To compound the difficulties of studying such children, neonatal screening raises major ethical concerns, and studies adopting this approach are very unlikely to be conducted in future.

More information is available on XXY because this karyotype affects endocrinological development and fertility in late adolescence and adulthood, and so hitherto unidentified cases come to attention.

A recommendation for future research is to ensure that there is a large comparison sample. It was frustrating to find that small groups of cases of SCT were often compared with equally small comparison groups, or sometimes no comparison group at all, when recruitment should not pose undue difficulty. It is not adequate to rely on test norms or clinical impression to identify neurodevelopmental deficits; a sizeable comparison group from a similar socio-economic background should always be included in a study. As illustrated in the studies reviewed here, comparison groups may be recruited from the same community, from siblings, or, as in one case, from children with a familial balanced autosomal rearrangement that did not affect the phenotype.

In general, research in this area has progressed from early somewhat anecdotal observations to studies placing greater reliance on standardized assessments, which allow one to quantify impairment in relation to a normative group, and to estimate error of measurement. However, this does not remove the need for information of a more descriptive and categorical kind, such as whether the child requires special help at school or will be able to live independently in adulthood, which may be of more importance to a parent than a test score. Here too, it is important to have a comparison group to interpret findings. Because IQ is often depressed in SCTs, IQ-matched comparison groups can be informative in establishing whether any problems are compatible with generally lower intellectual functioning or are a more specific correlate of the SCT. This approach was adopted in the Copenhagen sample^48^ and would be worth applying in future studies.

Work on the neurological bases of cognitive deficits in SCTs is still in its infancy, and there is a substantial imbalance in the published literature, with an almost exclusive focus on individuals with XXY, in contrast with very limited information on XXX and XYY. There is a paucity of studies of brain function rather than structure alone, which makes links to cognitive outcomes and difficulties rather speculative, and no researchers to date have investigated brain connectivity or neurotransmitter function. Furthermore, in the case of Klinefelter syndrome, studies of adults are complex to interpret, as there is evidence to suggest structural brain effects of testosterone treatment in males with XXY.^22^ As yet, nothing is known about the effects of dosage and duration of hormone treatment on brain structure in XXY.

There is also a need for more longitudinal data. The Denver, Edinburgh, and Toronto studies have all followed the same cases over time, but, probably because of small sample sizes and sample attrition, investigators have seldom considered how far early variables predict later outcomes in individuals. It would be interesting, for example, to consider whether a tendency to withdraw from novelty at the age of 3 years in males with XXY^43^ is predictive of psychosocial outcomes in adolescence or adulthood.^20^ Or to take another example, are anxiety and depression in adolescence predicted by poor social adjustment or academic failure?

Bender et al. have emphasized that future studies should be focused not only on the average outcomes of children with SCTs but also on individual variation and the causes thereof. In their sample they were able to show that children with SCTs seemed particularly sensitive to adverse environmental factors, whereas a supportive home background appeared to act as a protective factor.^16^ This contrasts with an analysis by Netley, who did not find any evidence of a protective effect of family background on either educational or behavioural problems in children with SCTs.^55^ Clearly, there is considerable scope for further work to identify the factors associated with optimal outcomes.

### Conclusion

The information collected so far on individuals with SCTs indicates that this is a vulnerable group at increased risk of educational failure and neurodevelopmental disorder. However, there is considerable variation in outcomes within each of the three groups reviewed here. Furthermore, the majority of children with SCTs will be able to live independently and form close relationships as adults. Nevertheless, these conclusions are based on a remarkably small evidence base. The limited data from unselected studies suggest varied outcomes, but considerable work needs to be done to chart and understand this variability, and to explain the risks and protective factors that may be associated with outcomes at the cognitive and neurological level.

Because of difficulties in recruiting participants, recent studies have tended to be focused on cases identified in childhood or adulthood via self-help groups or genetics clinics, and most of these have been restricted to Klinefelter syndrome. Although these studies may provide useful insights into neuropsychological profiles and brain correlates in individuals with an extra X chromosome, they can give a misleading picture of the typical outcome, as they will inevitably exclude individuals who are not giving cause for concern.

Neonatal screening is no longer feasible, but there is potential for studying larger groups of children identified on prenatal screening. Although such a sample is not totally unbiased – it is likely to contain a disproportionate number of older parents and will inevitably include only those parents who decided to continue the pregnancy – it could provide valuable information on typical outcomes for children with SCTs, without the bias that comes from selecting only those cases diagnosed because of childhood problems. A dilemma for such a study is that the child may not have been told about the trisomy. Nevertheless, one could use standardized methods of parental reporting, which have provided valuable information in some of the studies reviewed here and typically agree well with direct testing.

In addition to informing our understanding of brain–behaviour relationships in people with an extra sex chromosome, we urgently need more information about the impact of SCTs to guide clinicians as they advise parents receiving a prenatal diagnosis about possible future outcomes, and to set the scene for targeted intervention early in life, where appropriate.

## ONLINE MATERIAL

**Table SI:** Studies included in the review of sex chromosome trisomies (SCTs).

**Table SII:** Selection of comparison groups and areas assessed in the reviewed studies.
