# Supplementary material for: Neurocognitive outcomes of individuals with a sex chromosome trisomy: XXX, XYY, or XXY: a systematic review
Source: Dev Med Child Neurol. 2010 Jan 5;52(2):119–29. doi: 10.1111/j.1469-8749.2009.03545.x (PMC2820350; doi:10.1111/j.1469-8749.2009.03545.x)
Supplement: Supplementary file 1 [file dmcn0052-0119-SD1.doc]

**Table SI:** Studies included in the review of sex chromosome trisomies (SCTs).

**Table SII:** Selection of comparison groups and areas assessed in the reviewed studies.
